# Direct Effects of the Home, School, and Consumer Food Environments on the Association between Food Purchasing Patterns and Dietary Intake among Rural Adolescents in Kentucky and North Carolina, 2017

**DOI:** 10.3390/ijerph14101255

**Published:** 2017-10-21

**Authors:** Alison Gustafson, Stephanie Jilcott Pitts, Jordan McDonald, Hannah Ford, Paige Connelly, Rachel Gillespie, Emily Liu, Heather Bush, Candace Brancato, Toyin Babatande, Janet Mullins

**Affiliations:** 1 Department of Dietetics and Human Nutrition, 206J Funkhouser Building, University of Kentucky, Lexington, KY 40546, USA; jmc295@uky.edu (J.M.); Hannah.ford@uky.edu (H.F.); paige.connelly@uky.edu (P.C.); rlgi223@uky.edu (R.G.); janet.mullins@uky.edu (J.M.); 2 Department of Public Health, Brody School of Medicine, ECU 600 Moye Blvd., Mailstop 660, Lakeside Annex Modular Unit 8 Room 126, Greenville, NC 27834, USA; jilcotts@ecu.edu; 3 Department of Nutrition, University of Pennsylvania, College Station, PA 20067, USA; Emily.liu@gmail.com; 4 College of Public Health, University of Kentucky, 725 Rose Street Room 301, Lexington, KY 40536, USA; heather.bush@uky.edu (H.B.); candace.brancato@uky.edu (C.B.); 5 Health Sciences Bldg, 3310J MailStop #668, College of Allied Health Sciences, East Carolina University, Greenville, NC 27834, USA; Babatundeo@ecu.edu

**Keywords:** consumer food environment, school food environment, home food availability, adolescent obesity, nutrition

## Abstract

*Background*: Obesity rates are higher among rural versus urban adolescents. To examine possible mechanisms for the rural-urban adolescent obesity disparity, we examined the direct and indirect effects of food purchasing patterns, and the home, school, and consumer food environments on dietary intake among rural adolescents. *Methods*: A baseline survey was conducted among adolescents in eight rural high schools (four in Eastern Kentucky, and four in Eastern North Carolina). Participants answered questions about food purchasing patterns, dietary intake, home food availability, and demographics. The school and consumer food environments were assessed using validated measures from the School Meals Cost Study (United States Department of Agriculture-Mathematica) and the Nutrition Environment Measurement Survey for Stores, Restaurants, and Corner Stores. *Results*: Of 432 adolescents, 55% were normal weight, 24% were overweight, and 21% were obese. There was a direct association between unhealthy food purchasing patterns (shopping frequently at gas stations, fast food, and dollar stores) and consuming more added sugars, when compared to those with a healthy shopping pattern (shopping less frequently at gas stations, fast food, and dollar stores) [Odds Ratio = 2.41 (95% CI (confidence interval) 0.99, 3.82)]. Those who reported always having fruits and vegetables in the home consumed more servings of fruits and vegetables [OR = 0.31 cups (95% CI 0.22, 0.44)] compared to those who reported never having fruits and vegetables in the home. Adolescents attending a school with a low healthy food availability score consumed fewer servings of fruits and vegetables [−0.001 (95% CI −0.001, 0.0001)] compared to those attending a school with a high healthy food availability score. *Conclusions*: There are direct associations between food purchasing patterns, the home and school food environments, and dietary intake among rural adolescents. These cross-sectional results informed the development of the “Go Big and Bring it Home” program, a text messaging intervention to improve adolescents’ fruit, vegetable, and healthy beverage intake.

## 1. Introduction

Rural adolescents are more likely to be obese and eat fewer fruits and vegetables than their urban counterparts [1,2,3]. Neighborhood, socio-economic status, peers, the home food environment, and parenting styles affect adolescent dietary intake and obesity status [4,5]. However, less is known about factors that shape food purchasing habits among rural adolescents. It is especially important to focus on factors shaping food purchasing decisions at the critical juncture of adolescence (14–16 years of age), when youth are beginning to be more independent and are increasingly able to make decisions without the influence of parents or guardians [4]. However, there are few studies examining the association between the home, school, and consumer food environment, adolescents’ food purchasing patterns, and dietary intake.

Home food and beverage availability (types, quantity, and quality) influences adolescents’ consumption. For example, availability of chips and sweets in the home is associated with intake of such foods among children and adolescents [6,7], whereas home availability of fruits and vegetables is positively associated with fruit and vegetable consumption [8,9,10]. The school food environment may also affect adolescents’ dietary behaviors, as students in schools with healthier food-related policies have healthier diets compared to those with less healthy food-related policies [11]. Students, particularly those eligible for free and reduced lunch, consume the majority of their calories at school [12] and, thus, understanding how choices are associated with the school food environment is critical to improving health of rural adolescents. The consumer food environment influences dietary choices [13], yet the exact mechanism(s) by which the retail food environment influences dietary intake are not clear [14,15]. As prior work has shown that individuals purchase sugary beverages and snack foods at schools, gas stations, and convenience stores [16,17,18], it is not surprising that proximity to fast food and convenience stores is associated with higher body mass index (BMI) among adolescents [19,20].

**Figure 1:**
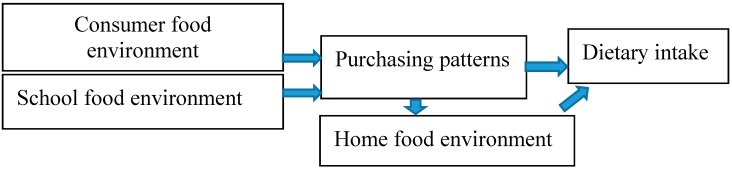
Conceptual model of pathways between food purchasing patterns, the home, school, and consumer food environments, and dietary intake.

Given the interdependent relationship between adolescents’ dietary intake and the food environment (home, school, and consumer), more work is needed to elucidate adolescents’ interactions with the home, school, and consumer food environments and dietary outcomes. Thus, the first purpose of this paper was to describe the sample of adolescents surveyed in Fall 2016, in order to inform the “Go Big and Bring it Home (GBBH)” intervention (a peer text message intervention to improve fruit, vegetable, and low-calorie beverage purchases), as shown in Figure 1. In addition, we sought to determine (a) the direct effect of food purchasing patterns on dietary intake; and (b) the potential mediation effect of the home, school, and consumer food environments on the relationship between food purchasing patterns and dietary intake.

We hypothesize that the consumer food environment (the foods and beverages available in retail food venues) influences what is purchased, which then influences dietary intake. We further hypothesize that food and beverage availability in the school influences what is purchased and consumed. Finally, food purchasing patterns in the community influence what is brought into the home, which influences dietary intake.

## 2. Materials and Methods

### 2.1. Sampling and Recruitment

Adolescents between the ages of 14–16 years living within four counties in rural Eastern Kentucky and four counties in rural Eastern North Carolina were recruited to participate in a baseline survey that led to the development of an intervention (“Go Big and Bring it Home”) to improve adolescent food and beverage choices in the home, school, and consumer food environments. In August–September 2016, adolescents received information that was sent home via various school channels (e-mail, newsletters, and home room announcements). Adolescents were also recruited from high school orientation sessions in August 2016. Eligibility criteria included the following: the adolescents must have resided in the county for at least one year, plan to reside in the county for at least one additional year, speak English as the primary language, and not report any serious illness that would alter their dietary patterns, such as diabetes or Crohn’s disease. If there was more than one adolescent in the household within the age range, both adolescents could participate. After expressing initial interest and meeting eligibility criteria, the adolescents’ parents/guardians completed the consent form, and the adolescent completed the assent form. Then the questionnaire was completed by the adolescent during school hours with the assistance of trained graduate students. In North Carolina, the questionnaire was completed during orientation sessions or at home, sometimes with assistance from parents. Questionnaires were checked for completeness. In North Carolina, questionnaires were shipped to Kentucky in batches for data entry and management using REDCap. The University of Kentucky (UK) Institutional Review Board (IRB) reviewed and approved the study, with East Carolina University deferring to the UK IRB as the IRB of record (16-0114-P4S).

### 2.2. Questionnaire

The questionnaire included demographic questions (age, race, sex), food purchasing patterns (foods and beverages purchased and location of purchases), perceived community food environment questions, and questions to assess the home availability of various types of foods and beverages. School and consumer food environment audits were completed to obtain objective characteristics of the school and consumer food environment. Each measure is described below.

### 2.3. Potential Mediating Variables—Home, School, and Consumer Food Environments

*Home Food Environment*—To ascertain availability of food within the home, Project EAT (University of Minnesota) validated questions were used [21,22,23]. Adolescents were asked whether certain food and beverage categories were available in the home (i.e., “Fruits and vegetables are available in my home”, “We have ‘junk food’ in my home”, and “Soda pop is available in my home”) with responses ranging from “never” to “always”. Based on the distribution of the data, the home food environment categories were collapsed and a multilevel variable was created which included the following categories: never available, sometimes available, and always available.

*School Food Environment*—To quantify the school food environment, a validated audit tool (created for use in the USDA School Nutrition and Meal Cost Study) was used among all eight high schools in October–November 2016 [24]. This audit includes several subscales and we used the competitive foods, vending, marketing around the cafeteria, and meal standards sub-scales ^33^. To quantify competitive foods, a checklist was completed in the school cafeteria during lunch hours, and ascertained the availability of the following items that would qualify as “competing” with the standard school lunch: chips, pretzels, fruit chews, and other a la carte items. A separate vending audit tool was used to capture the total number of vending machines in the school, the number that offered beverages and snack items, and the number of low or no calorie beverages (healthy) relative to full calorie (unhealthy) beverages in each beverage machine. For snack vending machines, auditors assessed the total number of healthy items (low calorie or low-fat) versus “unhealthy” items (high calorie, sugar, fat, or sodium). Lastly, marketing was assessed by whether fruit or vegetable posters or signs were available in the cafeteria or at check-out; whether fruits or vegetables were promoted in another way; and whether fruits or vegetables were available at check-out for purchase. Based on the total scores from each sub-scale, a summary score was used for “total healthy” and “total unhealthy” foods and beverages available and promoted in the school.

Two independent graduate research assistants completed training on the school environment audit forms, and independently completed audits. Discrepancies were resolved by consensus. For the purposes of this paper, the meal standards were not used due to no variation between sites, thus we incorporated marketing-related features (as described above), as this is the variable where there was the most variation. To derive the availability of healthy and unhealthy beverages and snacks, the following items from the audit tool were used: Healthy beverages consisted of milk, 100% fruit juice, diet soda, water, and low-calorie sports drinks. Unhealthy beverages consisted of regular full calorie soda, fruit drinks, sweet tea, lemonade, and regular calorie sports drinks. Healthy snacks consisted of baked chips, pretzels, fruit, low-fat granola bars or cereal bars, dried fruit, popcorn and nuts and seeds. Unhealthy snacks consisted of regular chips, regular granola bars, candy, fruit snacks, meat snacks, and baked goods. A raw count score was used for each category, such that the total number of healthy snacks, healthy beverages, a la carte items that were healthy, and marketing of healthy items was summed to create a final summary score of availability of healthy items. The same steps were taken for quantifying unhealthy items in the school food environment.

*Consumer Food Environment*—To quantify for the consumer food environment, the baseline questionnaire assessed the top three locations where students purchased foods and beverages. After data were collected and analyzed in Fall 2016 and Spring 2017, graduate research assistants completed the Nutrition Environment Measures in Stores, Restaurants, and Convenience Stores (all three tools: NEMS-S, R, and CR), in the supermarkets [25], convenience stores [26], and restaurants [27] where participants reported shopping most frequently. A consumer food environment score was calculated by summing the scores of quality, availability, and price from all food venues where audits were conducted. Each adolescent then received a summary score based on the food venues at which they reported shopping.

### 2.4. Independent Variable

*Food Purchasing Patterns*—As used in a prior study, the following questions were used to compute the food purchasing pattern variable:

(1) the food outlets where individuals purchased foods and beverages;
(2) frequency of shopping at each type of location (daily, weekly, monthly);
(3) amount spent in each type of location;
(4) types of food purchased (produce, packaged items, dry goods, frozen items, ready-prepared meals, snack foods, baked goods, dessert, candy).

One point was assigned for purchasing a healthy food or beverage, (24 food choices) and subdivided into categories of: beverage, snack, and fast food [28,29].

Cluster analysis was used as a data management tool to identify dietary purchasing clusters (using frequency of purchasing specific food and beverage items) and location clusters (using frequency of shopping at each type of food venue). Tree diagrams were further used to determine the number of distinct clusters; three mutually exclusive clusters were identified for both diet and location. Diet and food purchasing patterns were summarized for each cluster to identify the groups of healthy shoppers, moderately healthy shoppers, and unhealthy shoppers. These definitions were based on frequency of shopping (3 times or more per week, 1–2 times per month, less than once per month) at convenience stores, fast food restaurants, and other stores while addressing how often different food categories were purchased (Table 1). Supermarkets and supercenters did not cluster since all groups frequently shopped at these types of venues.

### 2.5. Dependent Variables

We used the National Health and Nutrition Examination Survey (NHANES) 2009–2010 questions to assess dietary intake of fruits, vegetables, added sugars (derived from foods such as pizza, tomato sauce, and sugar-sweetened beverages (SSB)). The NHANES questionnaire captures frequency in the following way: “How often do you consume fruits?”; “How often do you consume vegetables not including French fries or other fried potatoes”; “How often do you drink regular soda?” The response options are included below in Table 1 and show how frequency was converted to daily frequency. Predicted mean intakes and predicted probabilities were then calculated for the following categories: fruit and vegetables, added sugars, and sugar-sweetened beverages. These were the primary (fruit and vegetable) and secondary (added sugars and sugar-sweetened beverages) outcomes. NHANES developed scoring algorithms to convert screener responses to estimates of individual dietary intake. For probability of intake above or below specific thresholds, NHANES uses logistic regression to derive scoring equations to predict the probability of usual intake above or below specific thresholds. The daily frequency was then used as the dependent variable for each model.

### 2.6. Statistical Analyses

We used descriptive statistics to determine means and frequencies of demographics and food purchasing patterns and dietary intake. To model the direct association between the food purchasing patterns and dietary intake, multiple linear regression models were used, controlling for race, age, and sex. To model the mediation effect of the home, school, and consumer food environments on the relationship between food purchasing patterns and dietary intake, a seemingly unrelated regression model (sureg) was used to quantify the indirect effects of all three potentially mediating variables while controlling for race, age, and sex. The seemingly unrelated regression model utilizes the product of the coefficients method, finding the indirect effect of the mediating variables by multiplying the regression coefficients from the independent variable and the mediating variable, with the mediating variable on the dependent variable. This model was used due to the categorical nature of the independent variable, food purchasing patterns. All analyses were coded with Stata data analysis and statistical software version 12.0 (StataCorp LLC., College Station, TX, USA).

## 3. Results

Table 2 shows demographic characteristics, weight status, food shopping patterns, types of foods and beverages purchased, and availability of food within the home, school, and consumer food environments. The majority of students were white, with an average age of 15 years, and 55% were normal weight, whereas 45% were overweight or obese. Regarding the home food environment, 42% of adolescents indicated that fruits and vegetables were “never” available in the home. Less than one third (29%) indicated that “junk food” was never available in the home, and 35% reported soda was never available in the home. The school food environment (objectively measured through competitive (a la carte) foods, vending, and marketing audits) indicated that there were an average of 20 healthy beverages in vending machines and among competitive (a la carte) items. However, there were 51 unhealthy beverages available in vending machines and among competitive (a la carte) items. Additionally, there were nine healthy snacks available and 22 unhealthy snacks available. Lastly, a majority of food categories, both healthy and unhealthy, were purchased at supermarkets, and a large percentage of snacks and unhealthy beverages were purchased at convenience stores as well.

In assessing the direct association between food purchasing patterns, home, school, and consumer food environment, and dietary intake, we found statistically significant associations between an unhealthy food shopping pattern and higher intake of added sugars (Table 3). Those who frequently shopped at convenience stores, fast food restaurants, and other stores consume about 2.4 more teaspoons of added sugars [OR = 2.41 (95% CI 0.99, 3.82)] compared to those with a healthy food shopping pattern. Adolescents who reported having fruits and vegetables always available in the home consumed more total fruits and vegetables compared to those that reported never having fruits and vegetables in the home [0.31 (95% CI 0.22, 0.41)]. Additionally, those who reported always having junk food, chips, and candy in the home consumed more added sugars and fewer fruits and vegetables when compared to those that reported never having these food items in the home. Adolescents who attended a school with a higher score for total availability of healthy snacks and beverages consumed less sugar-sweetened beverages compared to those who attended a school with lower availability of healthy items. Lastly, adolescents who attended a school with unhealthier items consumed fewer fruits and vegetables compared to those who attended a school with more healthy items. There were no statistically significant direct associations between the consumer food environment and dietary intake (results available upon request). There were no significant indirect effects for the three mediating variables tested (home, school, and consumer) between food shopping patterns and dietary intake.

## 4. Discussion

Similar to a national study [18], the majority of rural adolescents in our sample reported frequently purchasing unhealthy beverages, snacks, and candy. We found a direct effect between home and school food availability and dietary intake among rural adolescents, similar to prior studies [4,30]. Lastly, the use of cluster techniques uncovered “shopping patterns” that were identified as unhealthy, moderately healthy, and healthy, and the unhealthy pattern was associated with a higher intake of sugars. Although the home and school food environments influence dietary intake, the types of stores where adolescents purchase foods and beverages is also related to dietary intake. This could be due to the foods and beverages available in those locales, and suggests the need for multi-level programs and policies to improve dietary intake among adolescents.

Our results indicate that, on average, there were more unhealthy beverages and snacks in schools relative to healthy options. This finding suggests that implementation of healthy food policies may not occur as intended. Since research suggests that availability of healthy food and beverage options within the school can influence dietary intake [11,12], more work is needed to understand what can facilitate implementation of federal, state, and district policies to improve healthy food and beverage availability within schools.

The clustering method allowed us to examine adolescents’ food purchasing patterns; further, we learned that unhealthy purchasing patterns are associated with dietary intake, namely, added sugars. Previous findings have indicated that adolescents who shop at convenience stores, gas stations, and fast food restaurants consume more SSB and less fruits and vegetables [18]. Our finding corroborates the previous results but also suggests that frequently shopping at these types of food venues is associated with dietary intake.

Taken together, our findings suggest the need for interventions that address the home, school, and consumer food environments, while simultaneously aiming to improve adolescents’ food shopping patterns. Findings described here informed the development of the “Go Big and Bring It Home (GBBH)” project, an intervention being implemented in Fall 2017, focused on improving food and beverage purchasing and consumption. The GBBH project targets the home, school, and consumer food environments, in order to positively influence food purchasing habits. Adolescents receive text messages from undergraduate students aimed to help them improve their food purchasing choices. They will receive text messages two times per week at pivotal time points at which to encourage healthy choices within the home, school, and consumer food environments.

Previous studies have found the home food environment mediates the association between adolescents’ SSB intake and parental eating patterns [31]. The current study did not find mediating effects of the home, school, and consumer food environments. However, this does not definitively indicate there is no mediation effect of these variables; rather, it could be that due to the small sample size of schools and the self-reported measure of the home food environment, potential mediators were impossible to detect. Future work could benefit from a larger number of schools and objective measures of home availability.

Limitations of this study include the cross-sectional study design: thus, associations do not infer causality. Self-report of dietary measures and food purchasing patterns (both from adolescents or their parents) may be biased—however, there were no significant differences between sex or age on dietary measures. Thus, there is no indication of systematic under- or over-reporting and no indication of misclassification bias. In some instances, North Carolina parents assisted students and, thus, responses among those students may be biased. This study did not assess built environment features, such as the number of stores in a neighborhood or type of stores within a specified boundary, and this limits our ability to discuss how neighborhood availability and access influence food purchasing patterns. However, given the large body of literature indicating the distal role that the neighborhood food environment has related to dietary intake, this study aimed to address more proximal determinants of dietary intake. Lastly, this sample is rural and, therefore, results cannot be generalized to urban populations.

## 5. Conclusions

These results led to the development of an intervention aiming to improve food shopping patterns and availability of healthy food items within the home, titled the “Go Big and Bring it Home” project. While the intervention will not directly change the school food environment or the consumer food environment, text messages will encourage adolescents to make smart food shopping choices while at home, school, and out in the community.

## Figures and Tables

**Table 1:** Food and beverage responses and conversion to frequency of derived daily intake.

Reported Frequency	Derived Daily Intake(oz)
Never	0
1–2 times/month	0.033
1–2 times/week	0.143
3–4 times/week	0.5
5 or more times/week	0.786

**Table 2:** Description of the study sample, adolescents ages 14–16 years in eight counties in Kentucky and North Carolina, 2017 (*n* = 432).

Demographics	Mean or Percentage
**Race**	
White	62%
Black	26%
Other	12%
**Average Age in Years**	15
**Gender**	
Female	41%
Male	59%
**Body Mass Index Categories**	
Normal (BMI 18–24.9 kg/m^2^)	55%
Overweight (BMI 25–29.9 kg/m^2^)	24%
Obese (BMI 30 kg/m^2^ and above)	21%
**Home Availability**	
Fruits and vegetables are available in my home (number, % never)	42%
Vegetables are served at home (number, % never)	32%
“Junk food” is available at home (number, % never)	29%
Potato chips/salty snack foods are available at home (number, % never)	29%
Chocolate/candy available at home (number, % never)	19%
Soda pop available at home (number, % never)	34%
**Food Purchasing Frequency (mean times per week food categories are purchased)**	
Fruits and Vegetables	11
Fast Food	12
Snacks	13
Healthy Beverages (low or no calorie drinks, milk, 100% fruit juice)	31
Unhealthy Beverages (sugar-sweetened beverages)	25
**School Availability (mean number of items available in vending and a la carte)**	
Healthy Snacks	9
Unhealthy Snacks	22
Healthy Beverages (low or no calorie drinks, milk, 100% fruit juice)	20
Unhealthy Beverages (sugar-sweetened beverages)	51
**Consumer Food Availability (range of scores with higher score indicating greater availability of healthy items possible highest score is 185)**	68–154
**Percentage of time food categories are purchased at the following locations**	
*Locations of Fruit and Vegetable Purchases*	
Supermarket	85%
Convenience Store	13%
School and Recreation Center	9%
Fast-Food Restaurant	4%
*Locations of Snack Purchases*	
Supermarket	76%
Convenience Store	40%
School and Recreation Center	13%
Fast-Food Restaurant	13%
*Locations of Healthy Beverage Purchases*	
Supermarket	72%
Convenience Store	46%
School and Recreation Center	15%
Fast Food Restaurant	16%
*Locations of Unhealthy Beverage Purchases*	
Supermarket	59%
Convenience Store	42%
School and Recreation Center	13%
Fast-Food	15%

**Table 3:** Association between food shopping pattern and food environments with daily dietary intake, North Carolina and Kentucky, 2016.

		Unhealthy Shopping Pattern	F/V Always in Home	Junk Food Always in Home	Chips Always in Home	Candy Always in Home	Soda Always in Home	School Healthy Score	School Unhealthy Score
dietary intake	F/V (cup)	0.06 (−0.08, 0.2)	0.31 (0.22, 0.41) *	−0.15 (−0.24, −0.06) *	−0.12 (−0.21, −0.33) *	−0.17 (−0.27, −0.07) *	−0.1 (−0.18, −0.01) *	−0.001 (−0.003, 0.001)	−0.001 (−0.001, −0.0001) *
	Added Sugar (tsp)	2.41 (0.99, 3.82) *	0.84 (−0.15, 1.84)	2.24 (1.36, 3.12) *	3.36 (2.51, 4.2) *	3.52 (2.61, 4.43) *	1.69 (0.85, 2.5) *	0.001 (−0.02, 0.02)	−0.0004 (−0.005, 0.004)
	Sugar Sweetened Beverages (tsp)	0.01 (−0.03, 0.06)	−0.02 (−0.05, 0.01)	0.01 (−0.01, 0.04)	0.01 (−0.02, 0.04)	0.01 (−0.02, 0.04)	0.01 (−0.02, 0.03)	−0.001 (−0.001, 0) *	−0.0001 (−0.0002, 0.0001)

* *p* < 0.05; reference healthy shopping pattern; reference never in the home.
